# Fibrous Dysplasia of the Clivus: Case Report and Literature Review

**DOI:** 10.7759/cureus.45417

**Published:** 2023-09-17

**Authors:** Veronica E Nkie, Sandra Martin

**Affiliations:** 1 Osteopathic Medicine-IV (OMS-IV), Alabama College of Osteopathic Medicine, Alabama, USA; 2 Radiology, Coosa Valley Medical Center, Alabama, USA

**Keywords:** polyostotic, monostotic, benign, craniofacial, clivus, fibrous dysplasia

## Abstract

Fibrous dysplasia is a benign, developmental bone disorder that causes fibrous replacement of normal skeletal tissue. This may lead to weakness, distortion, and tissue expansion. Fibrous dysplasia can occur anywhere in the body, including the craniofacial area. The clivus is a central skull bone formed by the bases of the sphenoid and occiput, respectively. The clivus is a rare, usually unrecognized, and seldom reported location for the development of fibrous dysplasia. Although fibrous dysplasia of the clivus (FDC) is usually discovered by incidental findings, it can sometimes present with clinical symptoms.

In this case, we discuss a 30-year-old male who presents to the emergency room with headaches, altered mental status, and a prior presentation of location-related symptomatic epilepsy. Magnetic resonance imaging depicted a mass in the clivus, low in signal on T1 and mildly hypointense on T2 imaging. Follow-up computed tomography (CT) imaging, as recommended, revealed the classic presentation of FDC. In this paper, we discuss the significance of this condition and the importance of thorough investigation to rule out differential diagnoses that may present with similar acute symptoms as this patient.

## Introduction

Fibrous dysplasia is a benign, congenital skeletal disorder that leads to fibroosseous and intramedullary bone lesions. It is a primary disease of the bone and can lead to weakening, expansion, and distortion of the affected bone [1]. It is a condition that occurs when skeletal tissue is replaced by fibrous tissue. Fibrous dysplasia usually affects single, individual bones, in which case the condition is termed monostotic, seen in the majority (75-80%) of cases, or it can affect several bones, in which case it is termed polyostotic. The latter presentation is usually seen in childhood and commonly involves the frontal, ethmoid, and sphenoid bones [2]. Fibrous dysplasia is usually identified in patients by age 30, with no demonstrable preference for a particular sex. It is a benign condition, with the adult presentation usually asymptomatic and discovered incidentally on imaging during the investigation of an unrelated condition. Commonly involved bones include the femur, ribs, long bones, and craniofacial bones [3].

The clivus is a bone situated at the base of the central skull. It is formed by the bases of the sphenoid (basisphenoid) and occiput (basiocciput). Although rare, craniofacial manifestations of fibrous dysplasia are usually identified in the clivus. These usually present with no symptoms and are discovered incidentally on imaging for unrelated reasons. Sometimes, however, the fibrous replacement of bony tissues can lead to fractures, neurovascular and soft tissue compression, cosmetic deformity, and intracranial extension [4].

We present the case of a 30-year-old male and discuss the symptoms, pertinent findings, management, and follow-up in this case. We also highlight and compare other cases of this condition and discuss the importance of ruling out other causes of the acute symptoms seen in this patient.

## Case presentation

History and physical

A 30-year-old man presented to the emergency room with a two-day history of headaches. On this day, the headaches had been accompanied by visual disturbances, and he had been sent by his primary care physician with this complaint, with the recommendation that he get some cervical imaging to further evaluate the possible causes of his symptoms.

The patient endorsed a history of headaches that had progressively worsened in the past few months. Attempts at alleviating his discomfort with ibuprofen had been only mildly helpful. There was no significant medical history. He did not drink, smoke, or indulge in any illicit drugs. The review of systems was positive for headaches and blurry vision but negative for all other symptoms.

On physical examination, the patient was alert and oriented to person, place, and time. Cranial nerve tests were intact, as well as musculoskeletal, strength, and range of motion (ROM). Pupils were equally responsive to light and accommodation. The remainder of the physical exam was largely non-contributory.

Diagnostic imaging

**Figure 1:**
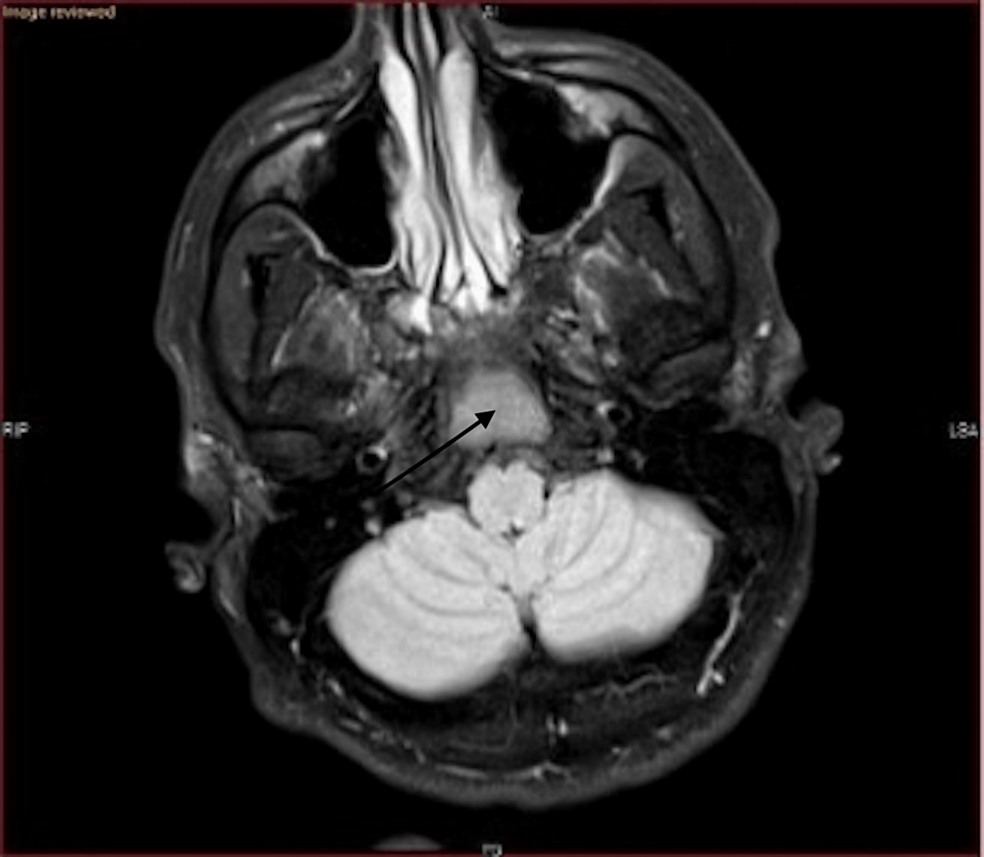
Mass in the clivus of low, hypointense signal intensity on T1-weighted image

**Figure 2:**
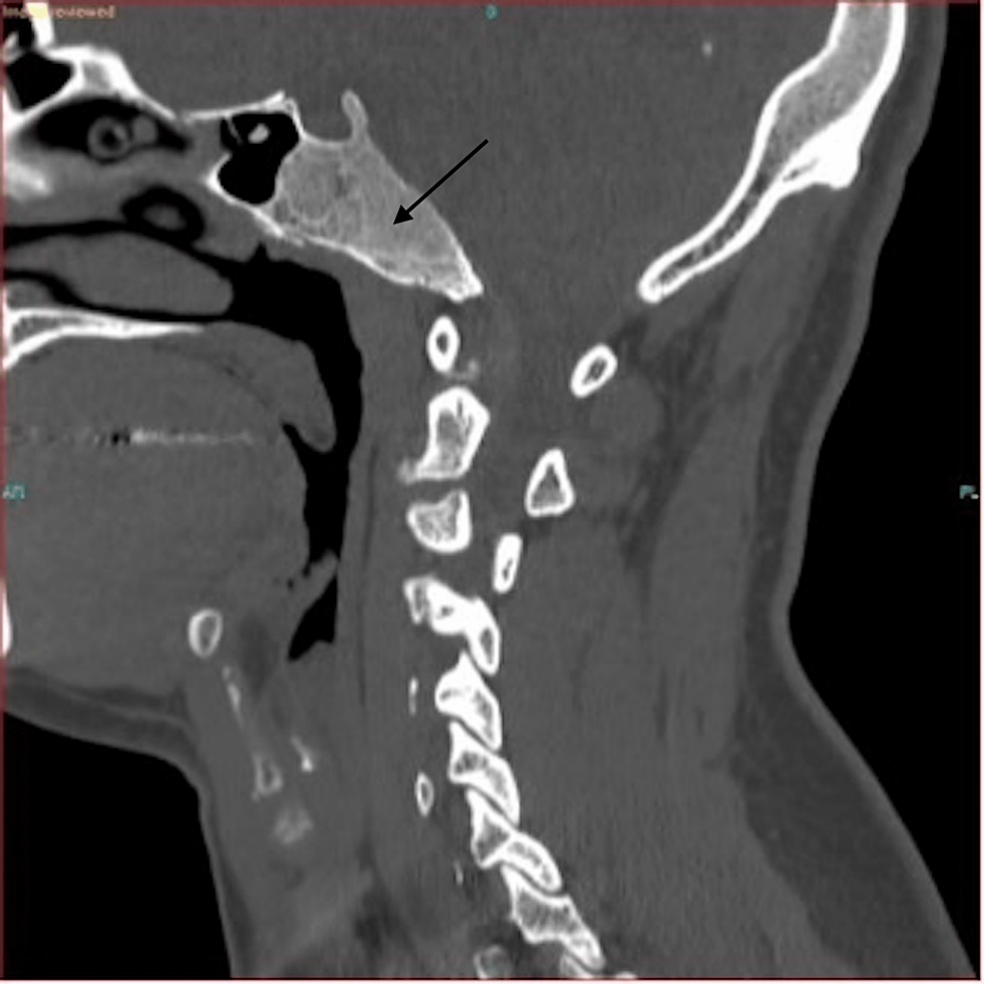
Mass in the clivus of low, hypointense signal intensity on T2-weighted image

In the emergency room, magnetic resonance imaging (MRI) of the brain without contrast was promptly ordered and performed. Figures 1-2 depict the MRI findings. According to the radiologist's report, "There is a mass in the clivus. This measures approximately 3.8 cm anterior-posterior, 2.2 cm craniocaudal, and approximately 2.3 cm transverse. This is low in signal on T1-weighted images and mildly hypointense in signal on T2-weighted images. I suspect it may reflect benign fibrous dysplasia. I recommend further evaluation with a CT scan of the skull base, as fibrous dysplasia often has a typical appearance on computed tomography (CT). No white matter signal abnormality is seen. There is no evidence of acute or subacute ischemia on the diffusion-weighted images. There is some mild ethmoid sinus disease seen, and there is frontal sinus consolidation (Figures 1-2)."

**Figure 3:**
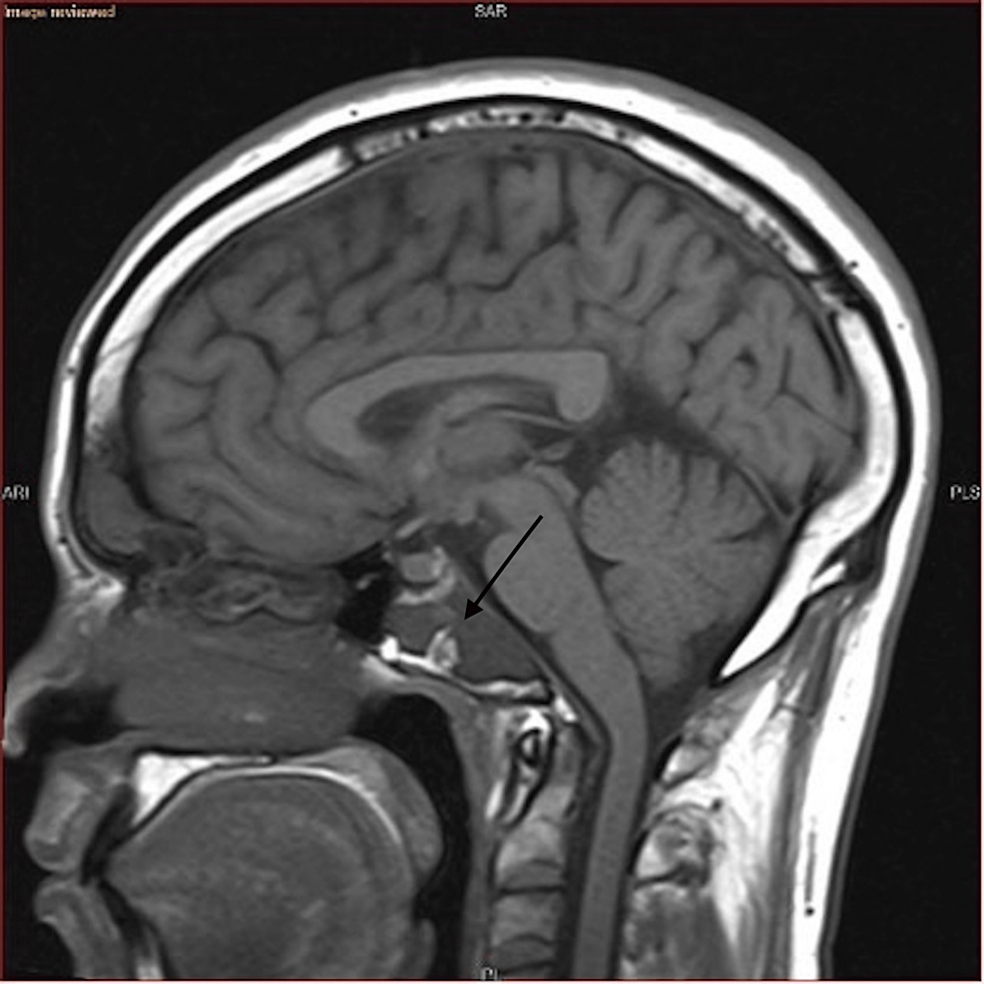
Ground glass appearance of clival mass is typical of fibrous dysplasia of the clivus

Following the radiologist’s recommendation, a CT scan without contrast was performed as well. The CT imaging result can be seen in Figure 3. The accompanying report described the findings as follows: "The expansile lesion seen in the clivus on the recent brain MRI has the typical ground-glass CT appearance of benign fibrous dysplasia (Figure 3)."

Management

Considering that our radiologist promptly identified this lesion as fibrous dysplasia of the clivus (FDC), it was recommended that no further lab tests or workup be performed in this patient. His treatment team, which included his internist, the emergency medicine physician, and his neurologist, concurred with the recommendation. Choices in management were discussed with the patient. These included watchful waiting with clinical observation and more frequent imaging given his symptoms, and elective surgery to relieve him of the lesion and possibly cure the symptoms and condition. 

## Discussion

Fibrous dysplasia is thought to be the result of a missense mutation in the GNAS 1 gene on chromosome 20. This leads to an activating mutation, leading to altered osteogenesis and abnormal proliferation of fibrous tissue [5]. It can be seen in McCune Albright syndrome, a rare condition of often unilateral polyostotic fibrous dysplasia with skin pigmentation and endocrine dysfunction manifesting as female precocious puberty. Another manifestation of fibrous dysplasia is Mazabraud syndrome, a very rare polyostotic variant seen independently or with concurrent intramuscular myxomas [6].

FDC is a relatively rare presentation of this disorder. A PubMed literature search identified 44 reported cases. Of these, one case was diagnosed with the polyostotic form, associated with McCune Albright Syndrome. In this case, the patient, diagnosed at age 3, developed severe complications secondary to McCune Albright, eventually succumbing to a subarachnoid hemorrhage at age 30. This arose due to the disruption of intracranial vasculature by severe bone disease [7]. A second case associated with McCune Albright was diagnosed in a six-year-old patient, with subsequent Chiari malformation and syringomyelia formation, whose spread was curtailed by surgery at age 20 [8]. A majority of the cases present as monostotic, and in adult patients, only one adult case presents a concurrent lesion in the thoracic spine [9].

Most cases of FDC present without symptoms. In symptomatic patients, the main presentation was headaches, either sudden or persistent, as seen in our patient. In the study by Ham et al., one patient presented with bifrontal headaches and disequilibrium for six months and another with supraorbital headaches and right facial hypesthesia for two weeks [10]. Other concurrent symptoms include limb weakness and cranial nerve symptoms with affected optic, olfactory, trigeminal, and auditory functions. Vertigo and tinnitus, non-tender occipital masses, and dysphagia have also been noted [11].

Considering that most patients present with no symptoms and this condition is usually discovered incidentally, a physical examination is usually non-contributory. In our patient, persistent headaches were the only symptom, and a physical exam proved unremarkable. In patients who present with symptoms, however, prompt follow-up testing and imaging are usually required to rule out differential diagnoses, some of which could prove debilitating or fatal without prompt intervention.

Imaging in fibrous dysplasia occurs with MRI and/or CT scans, scintigraphy, radiography, and MRI. MRI interpretation of the normal clivus denotes bright T1 fatty content relative to the pons. T1 hypointensity and T2 hyperintensity usually suggest a clival abnormality [12]. CT scans have been reported in some cases of fibrous dysplasia to depict ground glass opacity at the involved bones. Other descriptions have included cystic/lucent, sclerotic, or mixed densities. As such, densities and signal intensities will vary depending on the patient [13].

Given the presentation of fibrous dysplasia and its location in the clivus, a number of other conditions must be considered in the differential. Two important ones are chordomas and plasmacytomas, both of which can occur in the clivus. Intracranial chordomas are rare tumors thought to originate from remnants of the primitive notochord [14]. They are typically located at the skull base, or clivus, sacrum, or mobile spine. Similar to fibrous dysplasia in this location, they require MRI and/or CT for confirmation of diagnosis. On CT, chordomas appear as well-circumscribed soft tissue masses with extensive lytic bone destruction. The T1-weighted MRI images demonstrate hypointensity and can be clearly contrasted with the hyperintensity of normal clival fat. On T2-weighted MRI images, they demonstrate high signal intensity, likely secondary to high fluid content. Chordomas are aggressive tumors and usually require surgery and proton beam radiation therapy for optimal treatment [15].

Plasmacytomas are plasma cell tumors that arise within bone or soft tissue. They rarely occur in the skull and are usually located in the orbit, dorsum sellae, and sphenoid sinus [16]. They very rarely occur in the clivus, and when they do, they must be differentiated from other clival infiltrations like chordomas. Clival plasmacytomas tend to have a more rapid progression to multiple myeloma and a poorer prognosis compared to other intracranial forms [17]. Management of plasmacytomas is by radiation therapy, and surgery is only indicated when tumor bulk leads to neurologic issues secondary to the mass effect. Other clival tumors to consider include osteomas, chondromas, ameloblastic fibromas, and giant cell granulomas [18].

Treatment and management of FDC depend on the severity of the symptoms. Because the majority of cases are discovered incidentally and patients are generally asymptomatic, most patients are observed clinically. These lesions tend to be stable, and growth is not usually observed. However, malignancies have been reported in some cases, at almost 0.05% [19]. As such, patients are advised to follow up with yearly CT scans of the base of the skull in addition to clinical observation. Biopsies are not often required, and surgeries are reserved only for patients with progressive or debilitating symptoms such as severe headaches, vision loss, pain, or deformities. In these cases and in those in which patients desire removal of the lesion, the most widely used surgical approach is endoscopic endonasal transsphenoidal surgery. This is a minimally invasive procedure where the base of the skull is accessed via the transnasal route. Complete, subtotal, or partial resection is the goal, depending on clinical reasoning and the best outcomes for the patient [20].

## Conclusions

This case describes a 30-year-old male with fibrous dysplasia of the clivus, a rare cause of clival disease, and presentation symptoms secondary to mass effect. In this paper, we discuss the details of the case and review the existing literature. FDC is often discovered in asymptomatic patients as an incidental finding during imaging for other reasons. In a small subset of patients, FDC is discovered after symptoms develop, usually secondary to its location in the skull or mass effect. In asymptomatic patients, management is accomplished by clinical observation and follow-up imaging, usually yearly on average, as these are benign lesions and are not expected to become malignant. Patients with severe or debilitating symptoms are treated with surgery, with the most current approach being endonasal transsphenoidal resection.

In our case, after extensive discussions with the treatment team, our patient opted for close clinical monitoring and deferment of surgery. He was advised to return to the hospital if, at any point in time, he developed more serious symptoms such as worsening headaches, loss of vision, ataxia, paralysis, or weakness. He was discharged with medications for his headaches and scheduled follow-up dates for clinical evaluation and imaging.
